# Editorial: New Therapeutic Options for Rare Diseases

**DOI:** 10.3389/fped.2021.832395

**Published:** 2022-01-24

**Authors:** Sascha Meyer, E. Ann Yeh, Jürgen Brunner, Oliver Semler, Andrea Gropman

**Affiliations:** ^1^Department General Pediatrics, Neonatology, and Neuropediatrics, University Hospital of Saarland, Homburg, Germany; ^2^Division of Neurology, Department of Pediatrics, Neurosciences and Mental Health, SickKids Research Institute, Hospital for Sick Children, University of Toronto, Toronto, ON, Canada; ^3^Department of Pediatrics, Innsbruck Medical University, Innsbruck, Austria; ^4^Danube Private University, Krems, Austria; ^5^Department of Pediatrics, Faculty of Medicine and University Hospital Cologne, University of Cologne, Cologne, Germany; ^6^Neurogenetics and Neurodevelopmental Pediatrics, George Washington School of Medicine and Health Sciences, Washington, DC, United States

**Keywords:** rare diseases, children, pediatrics, diagnostics, new drug therapies, genetics, therapy

## Introduction

A rare disease is defined as a disease with <5 affected individuals in 10.000 people (1, 2). However, as a whole, rare diseases are not as rare as one might think. Rare diseases comprise roughly some 6,000–8,000 different clinical entities with genetic causes accounting for ~80% of rare diseases. In Europe, some 30 million people are estimated to be affected by a rare disease.

Due to advances in molecular diagnosis and early metabolic testing, many individuals are being diagnosed in infancy or early childhood. Of note, nowadays individuals with a rare disease may even be diagnosed *in utero*—as commonly seen in tuberous sclerosis complex disease (3). Nevertheless, in real life, it can often be quite challenging for affected people to find a physician with specific expertise in this field, thus leading to a delay in establishing a correct diagnosis and initiation of early, adequate treatment if available. Therefore, expert centers for rare diseases have been established by many university and tertiary hospitals. The aim of these centers is to create a network to facilitate diagnosis and optimize treatment and to generate and coordinate research activities. Treating rare diseases requires close cooperation between experts of various medical disciplines in a truly interdisciplinary setting (4). If a specific drug therapy is available, this is often associated with substantial costs for the health care system.

In this collection of up-to-date, cutting edge articles, several important aspects are addressed, most importantly novelties in diagnostics and new therapies with regard to rare diseases in children. In doing so, specific new insights into genetics, pathophysiology, and pharmacology are provided. While new promising therapeutic options are on the horizon (including genome editing), still many issues remain to be resolved, most importantly long-term outcome and survival. Thus, in addition to these new and potentially effective, individually tailored therapies, no definite cure can be provided for many children with rare and life-limiting diseases. Therefore, the role of palliative services must be stressed as well.

Due to the great number of rare diseases, this collection cannot be comprehensive in its coverage; it does, however, intent to shed light on new important and promising approaches for these highly vulnerable patients.

## Diagnostics

Li, Jia et al. describe two rare novel gross deletion mutations in the neurotrophic tyrosine kinase receptor type 1 gene (NTRK1) associated with congenital insensitivity to pain with anhidrosis in two unrelated families, employing whole exome sequencing, thus expanding the mutational spectrum of NRTK1 mutations. In their study, Qiu et al. identified 11 hub genes that may play critical roles in idiopathic pulmonary arterial hypertension (IPAH) by integrated bioinformatics analyses. The authors conclude that in addition to modifying the clinical course and progression of IPAH, they may also be candidate targets for IPAH treatment. Hu et al. report employed whole exome sequencing (WES) to describe a novel PAX3 mutation associated with Waardenburg syndrome type 1 (WS), thus expanding the understanding of (WS) Hu et al. Yang J.O. et al.also used whole-exome sequencing to provide new important insights into the spectrum of genetic variations associated with Lennox-Gastaut syndrome (LGS), a very severe type of childhood-onset epilepsy characterized by multiple types of seizures, specific discharges on EEG, and intellectual disability, in 17 unrelated Korean families by. In doing so, they were able to identify 14 mutations in 14 genes as causes for LGS or LGS-like epilepsy. Chen M. et al. report a case report with an unusual late-onset carnitine-acylcarnitine translocase deficiency with SLC25A20 c.199-10T>G variation, and they stress the importance of early recognition of symptoms and timely and appropriate treatment in improving outcome as mandatory for most rare diseases.

Murillo-Cuesta et al. for/on behalf of the Working group on Animal Models of Rare Diseases review examples of current advances in preclinical research in rare diseases using mouse models, and they discuss their perspective on future directions and challenges in this important diagnostic field.

Li L. et al. report on the role of high-throughput sequencing in revealing the loss-of-function mutation in GALT cause recessive classical galactosemia, thus expanding the phenotypic and mutational spectrum of GALT. Their findings could be helpful/informative in providing evidence for prenatal counseling/interventions and individually-tailored pharmacological treatments Li L. et al.

Tenembaum et al. describe the current state of knowledge with regard to clinical manifestations, diagnosis, and therapies for children with Neuromyelitis Optica Spectrum Disorders (NMOSD). They also provide insights with regard to the importance of auto-antibodies to aquaporin (AQP4-IgG) and myelin oligodendrocyte glycoprotein (MOG-IgG) in NMSOD (Tenembaum et al.) Chen Y. et al. studied the genetic and clinical features of Dopa-responsive dystonia (DRD) in 31 patients with DRD (1). Based on their results, DRD can be divided into classic DRD and DRD-plus disease. Interestingly, fever was the most important inducing factor of DRD, while L-Dopa exhibited sustained and stable effects in patients with classic DRD. In DRD-plus patients, treatment with L-Dopa ameliorated most of the clinical symptoms (Chen Y. et al.).

## Therapy

In their randomized-controlled study (28 patients: interventional group, 28 patients: control group), Wang et al.conclude that sirolimus may play a role in the treatment of systemic lupus erythematosus (SLE) by increasing the level of autophagy in peripheral blood lymphocytes. A major shortcoming, as in many clinical studies, was the fact that the authors did not assess long-term outcome in their study cohort (Wang et al.)

Liu et al. provide a systematic review on liver transplantation for glycogen storage disease type I (GSD) in 24 patients, a rare autosomal recessive disorder. While extra-hepatic manifestations, most importantly cardiac involvement may still progress, liver transplantation remains the only therapeutic option to increase both quantity and quality of life in children with GSD type 1 (Liu and Sun). Grant et al. report on cerebral revascularization surgery in a child with mucopolysaccharidosis type I, a rare lysosomal storage disorder, with overt stroke, infarction and bilateral terminal carotid artery stenosis with no further neurological events at a 3-year follow-up.

Flotats-Bastardas and Hahn present a succinct overview of new therapeutic options for patients with neuromuscular disorders, a genetically heterogeneous group of diseases. With the advent of new drugs in this field—either by ameliorating secondary pathophysiological consequences or by modifying the underlying genetic defect itself—treatment has changed fundamentally in recent years (5, 6).

Matsoukas provides a state-of-the art perspective on genome editing for rare genetic diseases without double-strand breaks or donor DNA, concluding that the development of prime editing provides a significant and important addition to the genome editing toolbox.

Sherzai et al. report on HMTase inhibitors as a potential epigenetic-based therapeutic approach for Friedreich's ataxia in FRDA fibroblasts, demonstrating that a combination therapy of G9a-inhibitor and EZH2-inhibitor [two histone methyltransferase (HMTase) inhibitor compounds] significantly increased frataxin (FXN) gene expression levels, but did not increase in frataxin protein levels.

Hully et al. provide on behalf of Neuromuscular Commission of the Societé Francaise de Neuropédiatrie the results from a prospective multicenter French study based on parents' reports about palliative care in 80 children with SMA type I (2012–2016). While their data confirmed previous reports on the natural history of this fatal disease, their study also demonstrated that palliative care has become an active approach involving a multidisciplinary team, and this has led to more home-hospital settings. The implementation of integrated palliative supportive care has played an important role in enabling more coordinated medical support (Hully et al.) Hully et al. also emphasize the importance of taking into consideration the advent of new drug therapies in patients with SMA with the potential to positively impact on life expectancy and quality of life. These new therapeutic avenues will also entail important medical, financial, and ethical issues. In that context, parents need to be clearly informed on the different therapeutic options with the remaining unknowns before they can consent to new treatment modalities (Hully et al.).

## Concluding Remarks

This article collection of the Research Topic provides the reader with an up-to-date overview on important progress in both diagnostic and therapeutic modalities in children with rare diseases. Despite new and promising diagnostic—probably most importantly the wide use of whole exome sequencing in clinical practice—and therapeutic avenues in these rare clinical entities, a definite cure for a substantial number of patients will not be at hand any time soon, thus stressing the importance of supportive and palliative care services (Hully et al.).

In the near future, it will be important to assess the efficacy of new drugs in high-quality clinical trials, including medium- and long-term outcome. To do so, robust epidemiological data with regard to incidence and prevalence of these rare diseases is of great importance (1–3). But, considering the very low incidence and prevalence of rare diseases, other important avenues to generate new and relevant data include both animal research models (Murillo-Cuesta et al.) as well as high-quality case reports as provided in this Special Issue (Chen M. et al.; Hu et al.; Grant et al.). Moreover, collaboration between experts throughout the world will have to be intensified.

We hope that this Special Issue on Rare Diseases in children will serve this purpose, and will be helpful in bringing together worldwide experts from many different fields (basic research, biology, genetics, biochemistry, medicine, nursing, palliative care, etc.), thus providing a basis to improve both diagnostic and treatment modalities for these very susceptible young patients.

## Author Contributions

This editorial was jointly conceptualized and written by all authors.

## Conflict of Interest

The authors declare that the research was conducted in the absence of any commercial or financial relationships that could be construed as a potential conflict of interest.

## Publisher's Note

All claims expressed in this article are solely those of the authors and do not necessarily represent those of their affiliated organizations, or those of the publisher, the editors and the reviewers. Any product that may be evaluated in this article, or claim that may be made by its manufacturer, is not guaranteed or endorsed by the publisher.
